# Stopping rules employing response rates, time to progression, and early progressive disease for phase II oncology trials

**DOI:** 10.1186/1471-2288-11-164

**Published:** 2011-12-12

**Authors:** John R Goffin, Greg R Pond

**Affiliations:** 1 McMaster University, Juravinski Cancer Centre, 699 Concession St., Hamilton, Ontario L8V 5C2, Canada; 2 McMaster University, Ontario Clinical Oncology Group (OCOG), Juravinski Hospital G(60) Wing. 1st Floor, 711 Concession Street, Hamilton, Ontario L8V 1C3, Canada

**Keywords:** early progressive disease, phase II trial, response rate, simulation, stopping rules, time to progression

## Abstract

**Background:**

Response rate (RR), the most common early means of assessing oncology drugs, is not suitable as the sole endpoint for phase II trials of drugs which induce disease stability but not regression. Time to progression (TTP) may be more sensitive to such agents, but induces recruitment delays in multistage studies. Early progressive disease (EPD) is the earliest signal of time to progression, but is less intuitive to investigators, To study drugs with unknown anti-tumour effect, we designed the Combination Stopping Rule (CSR), which allows investigators to establish a hypothesis using RR and TTP, while the program also employs early progressive disease (EPD) to assess for drug inactivity during the first stage of study accrual.

**Methods:**

A computer program was created to generate stopping rules based on specified error rates, trial size, and RR and median TTP of interest and disinterest for a two-stage phase II trial. Rules were generated for stage II such that the null hypothesis (*H*_nul_) was rejected if either RR or TTP met desired thresholds, and accepted if both did not. Assuming an exponential distribution for progression, EPD thresholds were determined based on specified TTP values. Stopping rules were generated for stage I such that *H*_nul _was accepted and the study stopped if both RR and EPD were unacceptable.

**Results:**

Patient thresholds were generated for RR, median TTP, and EPD which achieved specified error rates and which allowed early stopping based on RR and EPD. For smaller proportional differences between interesting and disinteresting values of RR or TTP, larger trials are required to maintain alpha error, and early stopping is more common with a larger first stage.

**Conclusion:**

Stopping rules are provided for phase II trials for drugs which have either a desirable RR or TTP. In addition, early stopping can be achieved using RR and EPD.

## Background

The goal of phase II clinical trials in oncology is to identify new drugs which are sufficiently promising in terms of efficacy to warrant further investigation [1]. By separating effective from ineffective treatments in phase II, appropriate phase III trials may be conducted. Efficient phase II trials designs are critical, as a large pool of drugs must be tested in a limited pool of patients at high cost [2,3]. However, just as there is typically uncertainty over the mechanistic specificity of new agents [4], phase II evaluation is complicated by uncertainty over what clinical outcomes might be observed and indicative of treatment efficacy. This renders the choice of clinical trial endpoints challenging, as some agents may induce tumour shrinkage, some may prevent worsening of disease, and others may do both, with variation by disease [5-8]. In addition, the majority of agents investigated in clinical trials are ineffective, and the ability to stop phase II trials early is desirable in such cases [3].

The most frequently employed phase II oncology endpoint is the response rate (RR) [9], which is most often defined using the RECIST criteria [10]. According to the RECIST criteria, a tumour response occurs if there is a 30% decrease in the sum of the longest diameters of measured tumour nodules. Tumour response has as its opposite progressive disease (PD), which is defined as a 20% increase in the same sum of diameters. Cases in which progressive disease occurs at the time of the first tumour measurement after treatment initiation can be termed early progressive disease (EPD). Tumours not shrinking or growing enough to reach definitions of response or progression are termed as having stable disease (SD).

Higher response rates are associated with improvements in survival [11-14] and are predictive of eventual regulatory approval [15], but this endpoint may not be appropriate for all drugs or diseases. Specifically, there are situations in which disease stabilization may occur but actual responses may be rare, such that using RR as the sole benchmark could lead to the dismissal of potentially useful drugs. For example, despite a response rate of 2%, sorefanib is now standard treatment for incurable hepatocellular carcinoma [8]. Phase III study only occurred because the failed phase II primary endpoint of response was ignored in favour of other signals of efficacy, including the duration of disease stabilization and survival [16].

Stable disease has also been associated with survival improvements [17], but is typically not used alone, rather frequently being combined with RR in an endpoint termed clinical benefit or disease control rate [18,19]. Alternatively, because a prolonged stable disease period would appear to offer patient benefit, other endpoints are used such as time to progression (TTP) [20], defined as the time interval until a cancer meets the definition of progressive disease. Progression-free survival (PFS) expands TTP such that the endpoint is marked at the time of either tumour progression or patient death.

Due to the large numbers of ineffective, and frequently toxic, agents studied in phase II, ethics dictate that many phase II clinical trials employ a two-stage method, which may be designed with the goals of minimizing trial size when agents are truly ineffective [21,22]. Using TTP or PFS rather than RR in a two-stage design generally requires a longer time to assess outcomes, potentially requiring additional patients to receive an ineffective treatment [23-25]. Furthermore, trials based on TTP or PFS alone may conclude an agent is inactive after stage I, even if it induces increased responses. In an untargeted population, it is possible that a subpopulation of patients of unknown size and an unknown molecular marker will have a tumour which is targeted by the treatment. Whether such tumours will shrink (i.e. respond) or simply stop growing (i.e. demonstrate an increased TTP) would be unknown. But there may be considerable interest in an agent which demonstrates either an increase in TTP, as occurred in the development of sorafenib, or RR, as was observed with crizotinib[26]. The ability to combine the RR and TTP endpoints would improve phase II trial sensitivity to drug activity when the nature of that activity is uncertain.

While much research has focused on RR and disease stabilization, the use of EPD has also been studied as part of a multinomial endpoint [27,28]. It should be noted that EPD is directly related to TTP, being by definition the earliest measurable manifestation of progression. If one assumes a common distribution for TTP in a population, a sufficiently high rate of EPD will predict a shorter (and perhaps undesirable) TTP. Yet, while EPD may provide an early signal of drug inactivity, it is not intuitive to clinicians, who are more accustomed to considering TTP comparisons using median values.

The present work combines endpoints with the aim of improving phase II trial sensitivity and specificity while addressing the need for intuitive measures. Specifically, the investigator specifies desirable and undesirable values for RR and TTP only, so that both potential manifestations of drug activity can be observed as signals of activity. The model then generates stopping rules for a two-stage trial with RR and TTP. In addition, employing an exponential distribution for progression in order to relate specified TTP parameters to their corresponding EPD values, the model generates stage I stopping rules using easily calculable RR and EPD rates. Using EPD at stage I in lieu of TTP avoids the delay required to observe TTP for the entire stage I cohort and allows earlier stoppage of the trial should EPD be too high (and therefore the corresponding TTP too low). This paper summarizes an assessment of this model using different parameters of interest to outline the possibilities and limitations of such a combined endpoint, hereafter termed the Combination Stopping Rule (CSR).

## Methods

Stopping rules for a single-arm, two-stage trial were constructed using simulations performed in TreeAge Pro Healthcare software (Version 1.0.2, 2009, Williamstown, Massachusetts) (program available on request). For this analysis, the desired statistical power and alpha error were restricted to ≥ 80% and ≤ 0.05 for the overall study throughout, however, other error limits could be used in the future as needed. For each simulation, the user specifies the RR of interest, RR of disinterest, median TTP of interest, median TTP of disinterest, and stage I and II sample size, (n_1_, n_2_). The user may also alter time of first tumour measurement and an absolute minimum median time for tumour progression allowable for a drug. Stopping rules are based on RR and median TTP at the second stage of accrual, but early stopping could occur at the end of the first stage of accrual when there are poor RR and EPD rates. Based on median TTP values of interest and disinterest, the model uses an exponential distribution to calculate EPD and assigns response as a dichotomous variable based on the specified probability.

The null hypothesis (*H*_nul_) specifies the response rate (*r*_nul_) and median TTP (*ttp*_nul_) that render a drug uninteresting for further development, such that: *H*_nul_: *r *≤ *r*_nul _and *ttp *≤ *ttp*_nul_, where *r *is that actual response rate and *ttp *is the actual median TTP. Similarly, the alternate hypothesis (*H*_alt_) specifies the response rate (*r*_alt_) and median TTP (*ttp_alt_*) that would render a drug interesting for further development, such that: *H*_alt_: *r *≥ *r*_alt _or *ttp *≥ *ttp*_alt_. At stage I, interpolating on the progression curve and using the time of first measurement to determine the resulting null EPD rate (*epd*_nul_), the null hypothesis is expressed as *H*_nul_: *r *≤ *r*_nul _and *epd *≥ *epd*_nul_, where *epd *is the rate of early progression, while the alternate hypothesis is expressed as *H*_alt_: *r *≥ *r*_alt _or *epd *≤ *epd*_alt_. Note that H_nul_, indicative of drug inactivity, is only accepted if both RR is low and median TTP is low (or at stage I, the surrogate of TTP, EPD, is high). At stage II, if either RR is high or median TTP is high, then H_nul _is rejected in favor of H_alt _and the drug is considered active. Early stopping at stage I for rejection of *H*_nul _is not permitted.

Functionally, using the investigator inputs, the simulation first establishes the stage II stopping rules (RR, TTP) required to achieve the desired power. The null hypothesis is rejected if *r*_1 _+ *r*_2 _≥ *r*_2a _or *ttp *≥ *ttp*_2a_, where *r*_1 _+ *r*_2 _is the cumulative number of patients with responses at the end of stage II, *ttp *is the median TTP at the end stage II, and *r*_2a _and *ttp*_2a _are the response and median TTP thresholds determined by the software. The stopping rules do not consider any association between the TTP value and response for an individual in the trial. The software then establishes stopping rules at stage I incorporating RR and EPD which optimize power at the expense of increased alpha error where necessary. At the end of stage I, therefore, the null hypothesis is accepted if *r*_1 _≤ *r*_1nul _and *epd *≥ *epd*_1nul_, where *r*_1 _and *epd *are the number of patients with response and EPD at the end stage I, and *r*_1nul _and *epd*_1nul _are the thresholds ascertained by the program.

Thresholds are identified by the program using 100,000 simulated trials. RR is evaluated using sequential increments of one patient, while for TTP increments are 0.25 months. For a threshold to be valid, it must satisfy the α error when RR = *r_nul _*and median TTP = *ttp_nul_*, and it must satisfy the β error when either RR = *r_alt _*or median TTP = *ttp_alt_*. For calculating the β error, half the simulated trials are performed with RR = *r_alt _*and median TTP randomly assigned to a value less than *ttp_alt_*, while the other half are performed with median TTP set to *ttp_alt _*and RR randomly assigned a value less than *r_alt_*. RR and EPD thresholds are then generated for the stage I test, while ensuring error rates are maintained for the entire study. Additionally, simulations are restricted such that RR + EPD ≤ 1 at stage I and by the imposed absolute minimum median time to progression.

The rate of patient censoring for median TTP estimation may also be altered by the user. For our modeling, it was assumed that patients who come off study due to toxicity or death (but not disease progression) prior to the time of first tumour measurement are replaced, although this may not be generalizable to all real-world phase II studies. Patients censored for TTP after the first tumour measurement were not replaced, and estimation of median TTP used the Kaplan-Meier method.

## Results

Thresholds generated by the software using a fixed sample size (n_1 _= 15, n_2 _= 15) while varying *H*_nul _and *H*_alt _are shown in Table 1. Parameters for *H*_nul _and *H*_alt _were based on the response values used in prior work [22,28] with the addition of plausible median TTP values. To interpret this table, the first row, where *r*_nul _= 0.05, *r*_alt _= 0.2, *ttp*_nul _= 3 and *ttp*_alt _= 6, would be read as follows: if there were zero responders and 5 or more patients with early progressive disease at the end of stage I, the study would be stopped and *H*_nul _accepted. Otherwise, the second stage sample would be recruited, after which *H*_nul _would be rejected if there were 5 or more responders or a median TTP of 5.25 months or higher. The resulting power would be 0.815 and the alpha error 0.035. For true uninteresting drugs, the probability of stopping the study (accepting *H*_nul_) at stage one would be 0.21, and the expected number of patients recruited would be 26.8.

**Table 1:** TTP and RR Thresholds Generated with fixed N while varying *H*_nul _and *H*_alt _(1-beta = 0.8, alpha = 0.05, censoring 0.05, n_1_ = 15, n_2_ = 15)

Response		Time to Progression		Stage 1 Drug Rejection		Stage 2 Drug Acceptance		Power	Alpha Error	EN_nul_ /PES_nul_	EN_alt_ /PES_alt_
*r*_nul_	*r*_alt_	*ttp*_nul_	*ttp*_alt_	*r*_1_	*epd*	*r*_1_+*r*_2_	*ttp*				
0.05	0.2	3	6	≤ 0	≥ 5	≥ 5	≥ 5.25	0.815	0.035	26.8/0.21	29.7/0.02
											
0.05	0.2	3	7	≤ 0	≥ 5	≥ 5	≥ 6	0.828	0.02	26.8/0.22	29.8/0.01
											
0.05	0.2	4	7	≤ -1	≥ 16	≥ 5	≥ 6.25	0.821	0.074	30/0	30/0
											
0.05	0.2	4	8	≤ 0	≥ 4	≥ 5	≥ 7	0.812	0.035	26.8/0.21	29.6/0.02
											
0.05	0.2	5	8	≤ 0	≥ 9	≥ 5	≥ 7	0.825	0.15	29.99†/ 0.0002	29.99†/ 0.0005
											
0.05	0.2	6	9	≤ 0	≥ 10	≥ 5	≥ 8	0.816	0.2	29.99/ 0.00001	29.99/ 0.0001
											
0.1	0.3	3	7	≤ 2	≥ 5	≥ 7	≥ 6	0.854	0.03	24.4/0.37	29.5/0.03
											
0.1	0.3	4	7	≤ 2	≥ 4	≥ 7	≥ 6	0.829	0.09	28.9/0.38	24.4/0.07
											
0.1	0.3	4	8	≤ 1	≥ 4	≥ 7	≥ 6.75	0.86	0.055	26.2/0.25	29.6/0.03
											
0.2	0.4	5	8	≤ 1	≥ 7	≥ 10	≥ 6.75	0.867	0.23	29.97/ 0.002	29.99/ 0.0004
											
0.2	0.4	5	9	≤ 3	≥ 3	≥ 10	≥ 7.5	0.813	0.13	24.6/0.36	28.6/0.09
											
0.3	0.5	5	8	≤ 4	≥ 3	≥ 12	≥ 6.75	0.828	0.29	25.7/0.28	28.4/0.1
											
0.3	0.5	6	9	≤ 4	≥ 3	≥ 12	≥ 7.5	0.843	0.35	26.7/0.22	28.7/0.09

†Truncated, not rounded, value.

EN_nul _= expected number of patients accrued if a drug meets the criteria of the null hypothesis

EN_alt _= expected number of patients accrued if a drug meets the criteria of the alternate hypothesis

PES_nul _= probability of stopping the trial after stage I if a drug meets the criteria of the null hypothesis

PES_alt _= probability of stopping the trial after stage I if a drug meets the criteria of the alternate hypothesis

For small studies (*n*_1 _= 15, *n*_2 _= 15), differentiating two endpoints is difficult, resulting in low probability of early stopping after stage I in some circumstances. In the most extreme case evaluated, a design with *r*_alt _= 0.2, *r*_nul _= 0.05, *ttp*_alt _= 7, and *ttp*_nul _= 4 results in stage I rejection values of *r*_1 _≤ -1 and *epd *≥ 16, indicating the study is unable to reject *H*_nul _at stage I and all trials will recruit 30 subjects. In other designs, the α error could not be maintained. Only trials with large differences between *r*_alt _and *r*_nul _as well as between *ttp*_alt _and *ttp*_nul _were able to satisfy both error estimates satisfactorily.

The effect of increasing the study size is seen in Table 2. Improvements in alpha error rates are observed and higher rates of early stopping are found. A minimally lower *ttp*_2a _is also sometimes noted, a result of the interplay between the thresholds chosen for RR and TTP; in larger studies, the model is able to find a value for *r*_2a _which gives a RR closer to *r*_alt _(i.e. higher), and the paired *ttp*_2a _is thus slightly lower to maintain the specified power. For studies with the highest *r*_alt_/*r*_nul _and *ttp*_alt_/*ttp*_nul _values, studies need to be relatively large to achieve an error rate of 0.05. Higher error rates may be acceptable in some circumstances.

**Table 2:** TTP and RR Thresholds Generated with Larger N (1-beta = 0.8, alpha = 0.05, censoring 0.05)

Response		Time to Progression		Study Size		Stage 1 Drug Rejection		Stage 2 Drug Acceptance		Power	Alpha Error	EN_nul_ /PES_nul_	EN_alt_ /PES_alt_
*r*_nul_	*r*_alt_	*ttp*_nul_	*ttp*_alt_	*n*_1_	*n*_2_	*r*_1_	*epd*	*r*_1_+*r*_2_	*ttp*				
0.05	0.2	3	6	30	15	≤ 2	≥ 8	≥ 7	≥ 5	0.863	0.015	36.6/0.56	44.5/0.04
													
0.05	0.2	3	7	30	15	≤ 2	≥ 7	≥ 7	≥ 6	0.849	0.008	35.0/0.67	44.4/0.04
													
0.05	0.2	4	7	30	15	≤ 2	≥ 7	≥ 7	≥ 6	0.85	0.037	38.3/0.45	44.4/0.04
													
0.05	0.2	4	8	30	15	≤ 2	≥ 7	≥ 7	≥ 6.75	0.864	0.014	38.3/0.44	44.6/0.03
												
0.05	0.2	5	8	30	30	≤ 2	≥ 6	≥ 9	≥ 6.75	0.874	0.06	47.7/0.41	58.5/0.05
												
0.05	0.2	6	9	30	50	≤ 2	≥ 5	≥ 13	≥ 7.75	0.84	0.063	58.4/0.43	76.5/0.07
												
0.2	0.4	5	8	30	30	≤ 6	≥ 6	≥ 20	≥ 6.75	0.872	0.075	50.8/0.31	58.5/0.05
												
0.2	0.4	5	9	30	40	≤ 7	≥ 6	≥ 23	≥ 7.5	0.888	0.019	54.5/0.39	68.2/0.05
												
0.3	0.5	5	8	30	50	≤ 7	≥ 6	≥ 34	≥ 6.75	0.888	0.053	65.2/0.3	77.1/0.06
													
0.3	0.5	6	9	30	70	≤ 9	≥ 5	≥ 43	≥ 7.5	0.864	0.073	72.9/0.39	93.2/0.1

If the censoring rate for TTP is increased to 0.1 from 0.05, the error rates and stage II thresholds are similar (Table 3). The stage I thresholds vary more in some cases.

**Table 3:** TTP and RR Thresholds Generated with Censoring set at 0.1

Response		Time to Progression		Study Size		Stage 1 Drug Rejection		Stage 2 Drug Acceptance		Power	Alpha Error	EN_nul_ /PES_nul_	EN_alt_ /PES_alt_
*r*_alt_	*r*_nul_	*ttp*_alt_	*ttp*_nul_	*n*_1_	*n*_2_	*r*_1_	*epd*	*r*_1_+*r*_2_	*ttp*				
0.05	0.2	3	6	15	15	≤ -1	≥ 16	≥ 5	≥ 5.5	0.812	0.034	30/0	30/0
													
0.05	0.2	3	7	15	15	≤ 0	≥ 5	≥ 5	≥ 6.25	0.822	0.021	26.8/0.21	29.8/0.01
													
0.05	0.2	4	7	15	15	≤ 0	≥ 4	≥ 5	≥ 6.25	0.811	0.084	26.8/0.21	29.5/0.03
													
0.05	0.2	4	8	15	15	≤ 0	≥ 10	≥ 5	≥ 7.25	0.818	0.038	29.998/ 0.0001	29.998/ 0.0001
													
0.05	0.2	5	8	15	15	≤ 0	≥ 9	≥ 5	≥ 7.25	0.821	0.151	29.996/ 0.0003	29.995/ 0.0004
													
0.1	0.3	3	7	15	15	≤ 2	≥ 5	≥ 7	≥ 6	0.864	0.033	24.4/0.37	29.5/0.03
													
0.1	0.3	4	7	15	15	≤ 2	≥ 4	≥ 7	≥ 6	0.837	0.106	24.4/0.38	28.9/0.08
													
0.1	0.3	4	8	15	15	≤ 1	≥ 4	≥ 7	≥ 7	0.859	0.054	26.2/0.25	29.6/0.03

In contrast to the Simon optimal or Fleming designs [22,29], the probability of early stopping (PES) of these designs appear to be reduced. For example, the Simon optimal design comparing r_nul _= 0.05 versus r_alt _= 0.20, with α ≤ 0.05 and β ≤ 0.20 and a total sample size of 29 patients, the PES after 10 patients is 0.599, while the Fleming design with 15 patients in each of 2 stages has a PES of 0.463, albeit with an α = 0.058. In contrast, the PES for the CSR is only 0.21, indicative of the increased difficulty of differentiating between two hypotheses.

## Discussion

Uncertainty over drug effect and the concern over discarding drugs that maintain disease stabilization without inducing tumour shrinkage has led investigators to look for alternatives to response rates as the sole marker of drug activity [30]. Recognizing this, the Combination Stopping Rule (CSR), which uses both median TTP and RR, is derived. The CSR incorporates EPD, based on estimates of TTP, in the stage I decision-making process to provide an early signal of drug inactivity and allow for early termination of an inactive agent.

Accepting the investigator's inputs for desirable and undesirable RR and median TTP, the model can generate thresholds for patient RR and median TTP for the second stage and patient RR and EPD rates for the first stage that meet the desired error rates. Larger studies are necessary to maintain acceptable alpha error rates when evaluating higher median TTP and RR values of interest.

Stopping rules employing RR only are well established and optimal designs have been proposed in terms of minimizing the number of patients required for study [22]. In the present study, values for *n*_1 _and *n*_2 _are specified by the investigator, making direct comparisons difficult. However, as the design measures two endpoints concurrently, the CSR generally requires additional numbers of patients in both stages, and greater levels of activity to deem a treatment of interest for further study [22,29]. The greater response requirement at stage II is a product of the CSR being designed to achieve the stated power when studying a population with an equal likelihood of having either 'good' response induction or 'good' time to progression.

In other work, EPD has been combined with RR [27,28]. That combination may change the sensitivity of the phase II trial to drug activity, stopping early to accept *H*_nul _in some additional instances and finding drug activity in some instances where the sole measurement of RR would not [31].

EPD and TTP each offer specific advantages. Compared with EPD, TTP is more intuitively meaningful to investigators, and it is easier to specify TTP durations of interest and disinterest when setting trial parameters. In addition, TTP is likely a better reflection of overall patient benefit than EPD, as EPD assesses only very early progression. Although trial sizes may be larger in some instances for the CSR than for those trials employing only RR or RR and EPD, this characteristic is common to studies assessing time to progression or progression-free survival [9,27,32]. Conversely, a disadvantage of TTP as a solitary endpoint is the time required to observe disease progression in sufficient numbers of patients. This can be particularly problematic for multistage trials, where holding recruitment at the end of the first stage to await results can negatively impact on recruitment momentum and cost. The CSR addresses this issue by interpolating back from the specified median TTP to create a stage I set of rules employing EPD. As such, the delay to stop an ineffective treatment at stage I is minimized. The present model therefore combines the familiarity of RR and TTP with the early signals of EPD measurement.

Stopping rules combining RR with TTP may be useful in the setting of targeted drugs with unknown clinical activity or in drugs which are believed to be cytostatic [33]. There is evidence that investigators are reluctant to rely upon response alone to measure new drug activity. In several studies where observed response rates have not achieved the predetermined threshold for activity, investigators have noted signals of disease stability or survival and advocated further study [34-37]. While imperfect, there is data to support a correlation between TTP and survival, and it may thus be a useful addition to RR alone [20,38].

There are limitations to the present study. The study employs TTP rather than PFS, while the latter is generally favoured because it includes survival [39]. Although rules adding PFS could be devised, they would require assumptions of a survival hazard in addition to assumptions about tumour growth and response, adding complexity to the model and uncertainty to the results. Similarly, randomization of phase II trials is recommended by some authors [40]. However, given the number of agents under investigation and the greater sample sizes required for randomized studies, non-randomized studies still predominate [9]. Furthermore, studies involving limited patient populations--such as those requiring an infrequent biomarker or rare disease--may render a randomized study impractical. Optimal single-arm methods are therefore still required.

Also, although the alpha error increases with smaller difference between *ttp*_alt _and *ttp*_nul_, practically, differences between *ttp*_alt _and *ttp*_nul _smaller than 3 months are unlikely to be interesting. It is noted too that the present study reports on only selected values for *n*_1 _and *n*_2_, although other values are possible. Finally, the stopping rules were generated with the assumption that a new drug under study has equal chances of having a desirable RR or a desirable TTP, although this cannot be known. Other assumptions could be made if it was felt that a drug was more likely to induce regression or stabilization, and the program could be modified.

As a model, the CSR cannot mimic disease processes with complete accuracy. The model assumes that the population undergoes tumour progression in an exponential distribution. It is unlikely that any one formula will adequately cover all diseases, and other curves, such as that of Gompertz, could be considered. However, exponential growth is a generally accepted distribution [41-43]. Testing the model with actual clinical trial data should provide insights into its behaviour. In addition, the model establishes actual tumour response independently from an individual subject's TTP within the study. This works for the model as responses are measured in aggregate, and responses could be assumed to be associated with the longer individual TTP's. This method was used for two reasons: first, it is unclear how a response should move a subject along the growth curve, and such a process would necessitate further assumptions. Second, the true median TTP of a simulated drug is established according to the investigator's input parameters and on whether true 'good' or true 'bad' drugs are being assessed. Allowing a response in an individual subject to influence that individual's growth curve (and thus TTP) requires that the TTP's of the remaining subjects be shifted in compensation, when such results should remain independent. Finally, the timing of tumour measurements during a trial will affect the trial's accuracy in detecting drug activity, a fact which needs to be carefully considered when using the CSR as well as other trial designs [44].

## Conclusion

The CSR provides a new method of measuring drug activity in a two-stage, phase II oncology trial by combining two well understood measures, RR and TTP. By also determining thresholds for RR and EPD at the first stage of accrual to assess for early signals of drug inactivity, the method allows for earlier stage I stopping without the delay that would be required by awaiting the TTP of every patient. This method is well suited to drugs which may have uncertain or low rates of response but which may induce stabilization.

## Competing interests

The authors declare that they have no competing interests.

## Authors' contributions

JRG designed the study, programmed simulations, analyzed data, and drafted the manuscript. GRP designed the study, analyzed data, and drafted the manuscript. Both authors read and approved the final manuscript

## Pre-publication history

The pre-publication history for this paper can be accessed here:

http://www.biomedcentral.com/1471-2288/11/164/prepub
